# Understanding Breast Cancer Awareness, Perceptions, and Screening Practices Among the Population of Jazan, Saudi Arabia: A Cross-Sectional Study

**DOI:** 10.7759/cureus.60759

**Published:** 2024-05-21

**Authors:** Ali Hendi, Jalal Abu Halimah, Naif Majrashi, Sarah Daghriri, Mohammed Alhafaf, Mohammed Alshaikh, Mohammed Akkam, Saleha Haroobi, Rahaf Othathi, Reem Harbi, Abdulrahman Zalah, Elham Maghrabi, Alanoud Masmali, Mohammed Mojiri

**Affiliations:** 1 Radiology, Jazan University, Jazan, SAU; 2 Surgery, Jazan University, Jazan, SAU; 3 Diagnostic Radiography Technology, Jazan University, Jazan, SAU; 4 College of Medicine, Jazan University, Jazan, SAU; 5 General Practice, King Fahd Central Hospital, Jazan, SAU

**Keywords:** saudi arabia, breast self-examination, mammography, screening practices, breast cancer

## Abstract

Background: Breast cancer represents a significant global health challenge, with Saudi Arabia experiencing high incidence rates, particularly among females. Early detection through screening methods such as mammography and breast self-examination offers promise in reducing mortality rates. However, participation in screening remains suboptimal, posing a barrier to effective cancer control. In regions like Jazan, situated in southwestern Saudi Arabia, comprehensive studies on breast cancer awareness and screening practices are lacking.

Methods: This cross-sectional study conducted in Jazan, Saudi Arabia, aimed to comprehensively assess breast cancer awareness, perceptions, and screening practices among the local population. An online survey platform was utilized to reach individuals aged 18 years or older residing in Jazan. Recruitment efforts utilized social media platforms, community networks, and local organizations to ensure diverse representation across socioeconomic backgrounds, education levels, and geographical locations. A meticulously designed questionnaire captured demographic information, breast cancer awareness, knowledge, health-seeking behaviors, screening practices, and barriers to mammogram screening. Participants provided electronic informed consent before self-administering the questionnaire.

Results: The study conducted in Jazan, Saudi Arabia, encompassed 533 participants, predominantly young to middle-aged individuals. Most participants were Saudi nationals (97.6%), employed in the government sector (55.7%), and resided in urban areas (61.0%). Awareness of breast cancer was high, with 98.1% having heard of the disease. However, perceptions of age of onset and prevalence varied. While participants showed varied awareness of breast cancer warning signs and risk factors, family history was a commonly agreed-upon risk factor (54.4%). Health-seeking behavior for breast cancer symptoms varied, with nipple changes prompting the most immediate medical attention (36.4%). Although most participants were aware of self-breast examination (84.6%) and mammograms (56.7%), utilization rates were suboptimal, with barriers including fear (79.7%) and embarrassment (71.5%) hindering mammogram screening uptake.

Conclusion: This study provides insights into breast cancer awareness and screening practices among participants in Saudi Arabia. While awareness of breast self-examination and mammography is high, disparities in screening service access persist due to barriers like fear and embarrassment. Addressing these barriers through culturally sensitive interventions and collaborative efforts is crucial for enhancing screening uptake and promoting health equity.

## Introduction

Breast cancer stands as one of the most pervasive and lethal cancers globally, exerting a significant toll on public health systems and individual lives. The World Health Organization (WHO) identifies female breast cancer as among the top three most common cancers worldwide, with a staggering 2.3 million cases reported in 2020 alone [1]. Tragically, breast cancer ranks as the leading cause of cancer-related deaths among females, claiming the lives of approximately 685,000 women globally in the same year [1]. Within the context of Saudi Arabia, breast cancer holds a particularly notable position, representing approximately 28% of all cancers affecting Saudi females and 13% of all cancers affecting both sexes in 2016 [2].

While breast cancer poses a formidable threat, early detection through screening tests such as mammography and breast self-examination offers a beacon of hope. The WHO underscores the potential of mammography screening in high-resource settings to reduce mortality by up to 20%, while early detection can dramatically elevate the chances of recovery to 90% or higher [3]. Despite the proven efficacy of screening modalities, participation rates among women remain below recommended levels, posing a formidable challenge to effective cancer control efforts [4].

In the Middle Eastern context, including Saudi Arabia, research on breast cancer awareness and screening practices has emerged, yet comprehensive studies assessing these aspects, particularly in regions like Jazan, remain scarce [5-7]. The Jazan region, nestled in the southwestern part of Saudi Arabia, presents a unique landscape characterized by diverse sociocultural dynamics and healthcare access challenges. Despite the prevalence of breast cancer in the region, there exists a notable gap in cross-sectional studies evaluating awareness and screening practices among the female population, particularly among key demographics such as female school teachers, who wield significant influence in disseminating health-related knowledge.

This study endeavors to bridge this gap by conducting a comprehensive assessment of knowledge, attitudes, and practices regarding breast cancer and radiological screening tests among female populations in Jazan City. Moreover, the research aims to identify factors influencing the uptake of breast cancer screening tests, thereby laying the groundwork for targeted interventions aimed at enhancing awareness and promoting proactive health-seeking behaviors.

## Materials and methods

Study design and setting

This cross-sectional study was conducted in Jazan, Saudi Arabia, with the aim of comprehensively assessing breast cancer awareness, perceptions, and screening practices among the local population. Data collection commenced in January 2024 and continued through an online survey platform for four weeks.

Participant recruitment

Participants were recruited using multifaceted approaches to ensure broad representation. Leveraging social media platforms, community networks, and local organizations, invitations to participate were extended to individuals aged 18 years or older residing in Jazan. The recruitment process emphasized inclusivity, targeting diverse socioeconomic backgrounds, educational levels, and geographical locations within the region.

Data collection instrument

A meticulously designed questionnaire was crafted to capture the nuanced aspects of breast cancer awareness, attitudes, and behaviors pertinent to the local context. Hosted on a user-friendly online survey platform, the questionnaire comprised multiple sections covering demographic information, breast cancer awareness and knowledge, health-seeking behaviors, screening practices, and barriers to mammogram screening. The instrument underwent rigorous piloting and validation procedures to ensure clarity, relevance, and cultural appropriateness for the study population.

Data collection procedure

Upon accessing the survey link, participants were presented with an electronic informed consent form outlining the study's purpose, confidentiality measures, and their rights as participants. Following consent, participants self-administered the questionnaire at their convenience, providing responses to the structured items. Data quality was ensured through validation checks within the online survey platform and double-data entry verification for online-based surveys.

Study variables

The study variables encompassed a wide range of demographic characteristics, including age, gender, education level, monthly income, occupation, nationality, residence type, and marital status, to capture the diverse sociodemographic profile of the population. Additionally, a comprehensive assessment of breast cancer awareness was conducted, exploring personal and family history of the disease, knowledge of the age of onset and prevalence, perceptions of risk factors and warning signs, immediate health-seeking behaviors for symptoms, awareness and practices related to screening methods such as self-breast examination and mammograms, and identification of barriers hindering mammogram utilization.

Data analysis

Data collected from the online survey platform were exported to a secure electronic database for analysis. Descriptive statistics, specifically frequencies and percentages, were computed using IBM SPSS Statistics version 25 (IBM Corp., Armonk, NY) to summarize demographic characteristics and study variables. These descriptive analyses provided insights into the distribution and composition of the study sample across categorical variables.

Ethical considerations

The study adhered to ethical principles and guidelines for research involving human participants. Institutional Review Board approval was obtained from the Saudi Oncology Health Economics Expert Group. Measures were implemented to safeguard participants' confidentiality, privacy, and voluntary participation throughout the study. Additionally, informed consent was obtained from all participants prior to their involvement in the study.

## Results

Demographic characteristics

The demographic profile of the study participants (N=533) revealed a predominantly young to middle-aged cohort. The majority fell within the age range of 20-34 years (379, 71.1%), followed by 35-45 years (101, 18.9%), and 46-55 years (49, 9.2%). Very few participants were aged 56-65 years (4, 0.8%). Regarding education, a significant proportion held a Bachelor's degree (362, 67.9%), followed by High School (126, 23.6%), Intermediate School (29, 5.4%), and Higher Education (16, 3.0%). Concerning monthly income, the distribution was relatively balanced, with 156 (29.3%) earning less than 5000 SAR, 162 (30.4%) earning 5000-10,000 SAR, 108 (20.3%) earning 10,000-15,000 SAR, and 107 (20.1%) earning more than 15,000 SAR. The vast majority of participants were Saudi nationals (520, 97.6%), primarily employed in the government sector (297, 55.7%) or as housewives (190, 35.6%). Residence-wise, the urban population slightly outnumbered the rural (325, 61.0% vs. 208, 39.0%). In terms of marital status, nearly half were single (257, 48.2%), followed closely by married participants (251, 47.1%), with smaller proportions being divorced (19, 3.6%) or widowed (6, 1.1%) (Table 1).

**Table 1:** Demographic profile of study participants (N=533) Data are reported in terms of frequency (n) and percentage (%). Frequency represents the number of participants in each category, while percentages indicate the proportion of participants in each category.

Characteristic	Category	Frequency (n)	Percent (%)
Age	20-34 years	379	71.1
	35-45 years	101	18.9
	46-55 years	49	9.2
	56-65 years	4	0.8
Education Level	Intermediate School	29	5.4
	High School	126	23.6
	Bachelor's Degree	362	67.9
	Higher Education	16	3.0
Monthly Income	Less than 5000 SAR	156	29.3
	5000-10,000 SAR	162	30.4
	10,000-15,000 SAR	108	20.3
	More than 15,000 SAR	107	20.1
Nationality	Non-Saudi	13	2.4
	Saudi	520	97.6
Occupation	Private Sector	46	8.6
	Housewife	190	35.6
	Government Employee	297	55.7
Residence	Rural	208	39.0
	Urban	325	61.0
Marital Status	Single	257	48.2
	Married	251	47.1
	Divorced	19	3.6
	Widowed	6	1.1

Breast cancer awareness and history

Among the study cohort, a negligible percentage reported personal breast cancer history (4, 0.8%), while a notable proportion had a family history of breast cancer (155, 29.1%). Almost all participants had heard of breast cancer (523, 98.1%), with varying perceptions of its age of onset and prevalence. Regarding age of onset, the majority believed it could occur at any age (188, 35.3%) or at 30 and above (190, 35.6%). However, a significant proportion was unsure (260, 48.8%) about the age of onset. Similarly, perceptions regarding breast cancer prevalence varied, with sizable proportions believing it to be one in nine (176, 33.0%) or one in three (108, 20.3%) (Table 2).

**Table 2:** Breast cancer awareness and family history (N=533) Data are presented as frequency (n) and percentage (%), representing the number and proportion of participants in each category, respectively.

Variable	Category	Frequency (n)	Percent (%)
Personal Breast Cancer History	Yes	4	0.8
	No	529	99.2
Family Breast Cancer History	Yes	155	29.1
	No	378	70.9
Heard of Breast Cancer	Yes	523	98.1
	No	10	1.9
Perceived Age of Onset of Breast Cancer	Any age	188	35.3
	Fifty and above	127	23.8
	Thirty and above	190	35.6
	Seventy and above	17	3.2
	Not sure	11	2.1
Perceived Breast Cancer Prevalence	One in 3	108	20.3
	One in 9	176	33.0
	One in 100	170	31.9
	One in 1000	79	14.8

Participants' perceptions of breast cancer warning signs

Participants demonstrated varied awareness regarding breast cancer warning signs. Axillary pain (325, 61.0%), palpable mass (308, 57.8%), and nipple discharge (258, 48.4%) were the most recognized symptoms. Conversely, nipple retraction (167, 31.3%), nipple lesion (177, 33.2%), and nipple dimpling (212, 39.8%) were less familiar to the participants. A considerable number were unsure about several symptoms, indicating potential gaps in knowledge (Table 3).

**Table 3:** Awareness and recognition of breast cancer warning signs (N=533) Data are presented as frequency (n) and percentage (%), representing the number and proportion of participants in each category, respectively.

Sign	Response	Frequency (n)	Percent (%)
Nipple Changes	Yes	194	36.4
	No	79	14.8
	Unsure	260	48.8
Nipple Retraction	Yes	167	31.3
	No	85	15.9
	Unsure	281	52.7
Axillary Pain	Yes	325	61.0
	No	39	7.3
	Unsure	169	31.7
Skin Changes	Yes	244	45.8
	No	57	10.7
	Unsure	232	43.5
Nipple Discharge	Yes	258	48.4
	No	49	9.2
	Unsure	226	42.4
Palpable Mass	Yes	308	57.8
	No	43	8.1
	Unsure	182	34.1
Axillary Mass	Yes	258	48.4
	No	59	11.1
	Unsure	216	40.5
Nipple Lesion	Yes	177	33.2
	No	73	13.7
	Unsure	283	53.1
Skin Dimpling	Yes	212	39.8
	No	73	13.7
	Unsure	248	46.5

Participants' perceptions of breast cancer risk factors

Concerning breast cancer risk factors, participants showed mixed opinions. The majority agreed or strongly agreed on family history (290, 54.4%), while uncertainty prevailed regarding other factors such as hormone replacement therapy (199, 37.3%), smoking (181, 34.0%), obesity (101, 18.9%), early puberty (46, 8.6%), late menopause (70, 13.1%), and exercise (54, 10.1%) (Table 4).

**Table 4:** Perceptions of breast cancer risk factors (N=533) Data are presented as frequency (n) and percentage (%), representing the number and proportion of participants in each category, respectively.

Risk Factor	Response	Frequency (n)	Percent (%)
Family History	Agree	199	37.3
	Strongly Agree	91	17.1
	Uncertain	158	29.6
	Disagree	32	6.0
	Strongly Disagree	53	9.9
Hormone Replacement Therapy	Agree	112	21.0
	Strongly Agree	42	7.9
	Uncertain	281	52.7
	Disagree	54	10.1
	Strongly Disagree	44	8.3
Smoking	Agree	181	34.0
	Strongly Agree	59	11.1
	Uncertain	190	35.6
	Disagree	53	9.9
	Strongly Disagree	50	9.4
Obesity	Agree	101	18.9
	Strongly Agree	23	4.3
	Uncertain	243	45.6
	Disagree	108	20.3
	Strongly Disagree	58	10.9
Early Puberty	Agree	46	8.6
	Strongly Agree	24	4.5
	Uncertain	230	43.2
	Disagree	130	24.4
	Strongly Disagree	103	19.3
Late Menopause	Agree	70	13.1
	Strongly Agree	24	4.5
	Uncertain	252	47.3
	Disagree	113	21.2
	Strongly Disagree	74	13.9
Exercise	Agree	54	10.1
	Strongly Agree	12	2.3
	Uncertain	188	35.3
	Disagree	122	22.9
	Strongly Disagree	157	29.5

Immediate health-seeking behavior for breast cancer symptoms

Participants' immediate health-seeking behavior for breast cancer symptoms varied. Nipple changes were the most commonly reported symptom, with 194 participants (36.4%) indicating seeking immediate medical attention. This was followed by nipple retraction (163, 30.6%), breast dimpling (162, 30.4%), and nipple discharge (256, 48.0%). Other symptoms prompting immediate attention included axillary mass (227, 42.6%), breast mass (222, 41.7%), nipple skin changes (182, 34.1%), breast redness (151, 28.3%), and change in breast shape (201, 37.7%) (Table 5).

**Table 5:** Prompt health-seeking behavior for breast cancer symptoms (N=533) Data are presented as frequency (n) and percentage (%), representing the number and proportion of participants in each category, respectively.

Symptom	Frequency (n)	Percent (%)
Nipple Changes	194	36.4
Nipple Retraction	163	30.6
Breast Dimpling	162	30.4
Nipple Discharge	256	48.0
Axillary Mass	227	42.6
Breast Mass	222	41.7
Nipple Skin Changes	182	34.1
Breast Redness	151	28.3
Change in Breast Shape	201	37.7

Breast cancer screening awareness and practices

Participants' awareness and practices regarding breast cancer screening varied. The majority had heard of the self-breast examination (451, 84.6%), with a significant number initiating the examination between the ages of 18 and 34 years (225, 42.2%). However, 280 participants (52.5%) reported never or rarely conducting self-exams. Regarding mammograms, most participants had heard of them (302, 56.7%), and the majority had access to mammogram centers (370, 69.4%). However, only a minority had undergone a mammogram previously (114, 21.4%), with 390 participants (73.2%) expressing willingness to undergo mammogram screening (Table 6).

**Table 6:** Knowledge and practices regarding breast cancer screening (N=533) Data are presented as frequency (n) and percentage (%), indicating the number and proportion of participants in each category, respectively.

Screening Activity	Response	Frequency (n)	Percent (%)
Heard of Self-Exam	No	82	15.4
	Yes	451	84.6
Self-Exam Initiation Age	13-17 years	132	24.8
	18-34 years	225	42.2
	35-44 years	63	11.8
	45-65 years	9	1.7
	Do not know	104	19.5
Self-Exam Frequency	Never or rarely	280	52.5
	Not sure	83	15.6
	Once per month	39	7.3
	Every 6 months	119	22.3
	Once per week	12	2.3
Self-Exam Confidence	Not at all confident	189	35.5
	To some extent	135	25.3
	Somewhat confident	169	31.7
	Very confident	40	7.5
Heard of Mammogram	No	231	43.3
	Yes	302	56.7
Availability of Mammogram Center	No	163	30.6
	Yes	370	69.4
Mammogram Initiation Age	Before age 40	258	48.4
	At or after age 40	275	51.6
Mammogram Cessation Age	Before age 75	253	47.5
	At or after age 75	280	52.5
Had Mammogram Before	No	419	78.6
	Yes	114	21.4
Willing to Do Mammogram	No	143	26.8
	Yes	390	73.2

Barriers to mammogram screening

Several barriers to mammogram screening were identified among participants. The most commonly reported barriers included fear (425, 79.7%), embarrassment (381, 71.5%), and anxiety (415, 77.9%). Other barriers included perception of time wastage (240, 45.0%), communication challenges (341, 64.0%), time constraints (348, 65.3%), appointment availability (350, 65.7%), transportation issues (336, 63.0%), and confidence concerns (320, 60.0%) (Table 7).

**Table 7:** Barriers to undergoing mammogram screening (N=533) Data are presented as frequency (n) and percentage (%), indicating the number and proportion of participants experiencing each barrier.

Barrier	Frequency (n)	Percent (%)
Embarrassment	381	71.5
Fear	425	79.7
Perception of Time Wastage	240	45.0
Communication Challenges	341	64.0
Time Constraints	348	65.3
Appointment Availability	350	65.7
Transportation Issues	336	63.0
Anxiety	415	77.9
Confidence Concerns	320	60.0

## Discussion

Our study sheds light on the landscape of breast cancer awareness, screening practices, and associated factors among participants in Saudi Arabia, offering valuable insights into the current state of breast health knowledge and behaviors in the region. Building upon existing literature, we highlight key findings that elucidate the prevalence of breast cancer awareness, disparities in screening uptake, and barriers to healthcare-seeking behavior among our study cohort.

The literature underscores the critical role of breast screening in detecting malignancies at early stages, thereby reducing morbidity and mortality rates associated with breast cancer [8-10]. Previous studies have highlighted the efficacy of healthcare provider-led initiatives in promoting routine breast examinations and increasing awareness of self-breast examination, clinical breast examination, and mammography [11]. Our study aligns with these findings, revealing a substantial awareness of breast self-examination and mammography among participants. However, despite widespread awareness, disparities persist in access to screening services, echoing findings from studies conducted across different cultural contexts [12].

Despite the availability of screening modalities, various barriers hinder screening uptake and healthcare-seeking behavior. Fear, embarrassment, privacy concerns, and lack of confidence emerge as significant impediments to timely screening and healthcare utilization, consistent with findings from studies conducted across different cultural contexts [13,14]. These findings underscore the need for culturally sensitive interventions and patient-centered approaches to address barriers and enhance screening adherence.

Healthcare professionals play a pivotal role in promoting breast cancer awareness and facilitating informed decision-making among patients. The literature emphasizes the importance of provider-patient communication, patient education, and community engagement in improving screening uptake and health outcomes [15]. Our study underscores the need for concerted efforts to equip healthcare professionals with the knowledge and resources to effectively communicate with patients and address misconceptions surrounding breast cancer screening.

Moving forward, future research should prioritize addressing knowledge gaps, enhancing access to screening services, and evaluating the effectiveness of interventions tailored to specific population groups. Longitudinal studies are warranted to assess the impact of educational campaigns, policy initiatives, and structural interventions on screening behaviors and breast cancer outcomes [15,16]. Moreover, collaborations between stakeholders, including healthcare providers, policymakers, advocacy groups, and community organizations, are essential to developing comprehensive breast cancer control programs and promoting health equity.

Despite the valuable insights gained from our study, several limitations warrant acknowledgment. First, the sample size may limit the generalizability of our findings to broader populations, particularly across diverse demographic groups. Secondly, the gender composition skewed heavily toward females, potentially impacting the representativeness of our study population. Additionally, the majority of participants were under the age of 40, which may have influenced the observed screening behaviors and awareness levels, particularly regarding mammography. Furthermore, the reliance on self-reported data introduces the possibility of recall bias. Future research endeavors should aim to address these limitations by employing larger, more diverse samples and utilizing objective measures of screening behavior and awareness.

## Conclusions

In conclusion, our study provides valuable insights into the landscape of breast cancer awareness and screening practices among participants in Saudi Arabia. Despite a notable awareness of breast self-examination and mammography, disparities in access to screening services persist, exacerbated by barriers such as fear, embarrassment, and lack of confidence. Moving forward, concerted efforts are needed to address these barriers and enhance screening uptake through culturally sensitive interventions and patient-centered approaches. Collaborative endeavors between healthcare providers, policymakers, advocacy groups, and community organizations are essential to developing comprehensive breast cancer control programs that promote health equity and improve outcomes for individuals across diverse demographic groups. By leveraging these insights and implementing evidence-based strategies, we can strive toward reducing the burden of breast cancer and improving the overall health and well-being of individuals in Saudi Arabia and beyond.
